# In this month’s Bulletin

**DOI:** 10.2471/BLT.25.000925

**Published:** 2025-09-01

In the editorial section, Mercedes Bonet et al. (518) describe implementation of WHO recommendations for antenatal, intrapartum and postnatal care. Jakob Zinsstag et al. (519) make the case for rabies elimination in the African Region. 

## Angola, Burundi, Cameroon, Chad, Democratic Republic of the Congo, Gambia, Ghana, Guinea, Kenya, Lesotho, Malawi, Mali, Namibia, Rwanda, Senegal, Sierra Leone, South Africa, Togo, United Republic of Tanzania, Zambia, Zimbabwe 

### Deaths from diarrhoea, pneumonia and measles

Stéphane Verguet et al. (522–529) review the data on inequalities in child deaths.

## Bangladesh, Botswana, China, Ethiopia, India, Indonesia, Kazakhstan, Mexico, Philippines, Russian Federation, Senegal, South Africa, Ukraine, United Republic of Tanzania, Viet Nam

### Cigarettes over a lifetime

Mark Goodchild et al. (541–549) estimate smokers’ total consumption.

## Benin, Malawi, Uganda, United Republic of Tanzania

### Caesarean sections and stillbirths

Maria del Rosario Alsina et al. (550–562) question surgical management.

## Gambia, Kenya, Mozambique

### Hypertension prevalence estimates

Laura A Magee et al. (563–569) quantify differences between reported and measured hypertension.

## Zambia

### Next steps in cervical cancer screening 

Ahmad Fuady et al. (530–540) compare costs of three treatment methods. 

## Global

### Nutrition labelling of food packaging

Katherine Shats et al. (570–573) discuss policy implications.

### Preventing racial discrimination in health care

Michal Balcerzak and Ewa Michalkiewicz-Kądziela (574–576) present a human rights approach.

